# Relationship between self-reported dietary intake and physical activity levels among adolescents: The HELENA study

**DOI:** 10.1186/1479-5868-8-8

**Published:** 2011-02-06

**Authors:** Charlene Ottevaere, Inge Huybrechts, Laurent Béghin, Magdalena Cuenca-Garcia, Ilse De Bourdeaudhuij, Frederic Gottrand, Maria Hagströmer, Anthony Kafatos, Cinzia Le Donne, Luis A Moreno, Michael Sjöström, Kurt Widhalm, Stefaan De Henauw

**Affiliations:** 1 Department of Public Health, Ghent University, Ghent, Belgium; 2 Inserm U955, IFR114/IMPRT, Faculty of Medicine, University Lille 2, F-59037 Lille, France; 3 CIC-9301-CH&U-Inserm of Lille, CHRU de Lille, F-59037 Lille, France; 4 Department of Physiology, School of Medicine, Granada, Spain; 5 Department of Movement and Sport Sciences, Ghent University, Ghent, Belgium; 6 Department of Biosciences and Nutrition, Karolinska Institutet, Sweden; 7 University of Crete School of medicine, Greece; 8 National Research Institute on Food and Nutrition, Rome, Italy; 9 Growth, Exercise, Nutrition and Development (GENUD) research Group, E.U. Ciencias de la Salud, Universidad de Zaragoza, Zaragoza, Spain; 10 Medical University of Vienna, Austria

## Abstract

**Background:**

Evidence suggests possible synergetic effects of multiple lifestyle behaviors on health risks like obesity and other health outcomes. Therefore it is important to investigate associations between dietary and physical activity behavior, the two most important lifestyle behaviors influencing our energy balance and body composition. The objective of the present study is to describe the relationship between energy, nutrient and food intake and the physical activity level among a large group of European adolescents.

**Methods:**

The study comprised a total of 2176 adolescents (46.2% male) from ten European cities participating in the HELENA (Healthy Lifestyle in Europe by Nutrition in Adolescence) study. Dietary intake and physical activity were assessed using validated 24-h dietary recalls and self-reported questionnaires respectively. Analyses of covariance (ANCOVA) were used to compare the energy and nutrient intake and the food consumption between groups of adolescents with different physical activity levels (1^st ^to 3^rd ^tertile).

**Results:**

In both sexes no differences were found in energy intake between the levels of physical activity. The most active males showed a higher intake of polysaccharides, protein, water and vitamin C and a lower intake of saccharides compared to less active males. Females with the highest physical activity level consumed more polysaccharides compared to their least active peers. Male and female adolescents with the highest physical activity levels, consumed more fruit and milk products and less cheese compared to the least active adolescents. The most active males showed higher intakes of vegetables and meat, fish, eggs, meat substitutes and vegetarian products compared to the least active ones. The least active males reported the highest consumption of grain products and potatoes. Within the female group, significantly lower intakes of bread and cereal products and spreads were found for those reporting to spend most time in moderate to vigorous physical activity. The consumption of foods from the remaining food groups, did not differ between the physical activity levels in both sexes.

**Conclusion:**

It can be concluded that dietary habits diverge between adolescents with different self-reported physical activity levels. For some food groups a difference in intake could be found, which were reflected in differences in some nutrient intakes. It can also be concluded that physically active adolescents are not always inclined to eat healthier diets than their less active peers.

## Background

Physical activity (PA) and dietary habits might have a strong influence on health. These two lifestyle habits are considered as fundamental risk factors for several chronic diseases, such as type 2 diabetes, cardiovascular diseases, obesity, etc. [1,2]. Also, the increasing prevalence of obesity in youth is considered a result of an imbalance between energy intake and energy expenditure [3]. Furthermore, obesity in young people is accompanied by a high risk to persist into adulthood [4]. This could be partly due to the fact that unhealthy lifestyle behaviours, such as poor diets and a lack of PA, have the tendency of tracking from childhood into adulthood [5,6]. Previous research using subjective measures of PA [7] has shown that only 12% to 42% of 13 year olds and 8% to 37% of 15 year olds achieved the PA recommendation to participate in moderate to vigorous PA (MVPA) for at least 60 min per day [8]. Furthermore, according to objectively measured data [9], fewer 15 year olds achieved the current activity recommendation compared to 13 year olds, a trend particularly noticeable in girls. It has also been indicated by the HBSC (Health Behaviour in School-aged Children) study [7,10] that adolescents have poor dietary habits, which are characterized by a high consumption of sweets and soft drinks, breakfast skipping, and a low consumption of fruits and vegetables. Changing those modifiable behaviors into healthy lifestyles, starting from childhood, has the potential to reverse the obesity epidemic and have important short and long term health protective effects. Therefore, the prevention of obesity and the promotion of healthy lifestyle habits in youth are high priorities.

According to Pronk et al. [11], there is also a possible synergetic effect of multiple health behaviors on the risk of chronic conditions and other health outcomes. Therefore, creating a better insight in the relationship between nutrition and PA and the co-occurrence of low physical activity and unhealthy dietary habits among adolescents are important before developing effective promotion and prevention strategies to enhance both health behaviors. Previous studies revealed associations between dietary habits and time spent in PA among adolescents. Kremers et al. [12] showed that a low frequency of fruit consumption clustered with spending little time in PA among Dutch adolescents. Others [13,14] have indicated that a high percentage of adolescents fail to meet the recommendation for fruit and vegetable intake (5 or more servings per day) [15] together with those for PA. This would mean that healthy dietary and PA behaviors often do not occur in isolation and are correlated with each other. A study by Kelishadi et al. [16] also revealed that the most active adolescents consumed fruit and vegetables and dairy products more frequently compared to their peers spending less time in PA. Furthermore, Coulson et al. [17] reported that a greater consumption of fresh foods was associated with a higher PA participation among English adolescents. On the other hand, inconsistent results were found in adult studies examining the difference in energy and nutrient intake between active and inactive persons [18]. Until now, no studies have examined the relationship between dietary intake, with the attention on both the nutrient and food group level, and self-reported PA levels among adolescents. Therefore, the aim of the present study was to describe the relationship between energy, nutrient and food intake and the PA level among a large group of European adolescents.

## Methods

### Study design

Data was derived from the HELENA-CSS (Healthy Lifestyle in Europe by Nutrition in Adolescence-Cross Sectional Study), which is a multi-centre study on lifestyle and nutrition among adolescents conducted in 10 European cities (Athens in Greece, Dortmund in Germany, Ghent in Belgium, Heraklion in Crete, Lille in France, Pecs in Hungary, Rome in Italy, Stockholm in Sweden, Vienna in Austria, and Zaragoza in Spain) [19]. The main objective was to obtain reliable and comparable data of a sample of European adolescents (12.5 - 17.5 years) on a variety of nutrition and health related parameters, such as PA, via standardized procedures [19]. Data collection took place from 2006 to 2007. A random cluster sampling of 3000 adolescents (based on random selection of classes), stratified for geographical location, age and socioeconomic status, was carried out. A list of 10 schools was provided to each centre and in case of refusal, a second list was already foreseen. A class was seen as eligible if the participation rate was at least 70%. If the participation rate was lower than 70%, the class was replaced by another one in the same age group in the same school. Further details on sampling procedures and study design of the HELENA study have been reported elsewhere [19]. The study was approved by the Ethical Committee of each city involved [20]. A written informed consent was obtained from both the adolescents and their parents for publication of this case report and accompanying images. A copy of the written consent is available for review by the Editor-in-Chief of this journal.

### Measurements

#### HELENA-Dietary Assessment Tool (HELENA-DIAT)

To obtain dietary intake data, the HELENA-DIAT 24-h dietary recall software was used. This 24-h recall assessment tool is based on six meal occasions referring to the day before the interview. The adolescents completed the questionnaire during school time, after dieticians/researchers instructed them on how to fill in this 24-h recall as accurately as possible. The participants were allowed to ask questions and assistance and after completion, the recall was checked for completeness. Every participant was asked to fill in the HELENA-DIAT on arbitrary days, twice in a time-span of 2 weeks. Since the questionnaire was filled in during school time, no data could be collected about the dietary intake on Fridays and Saturdays. A validation study by Vereecken et al. [21] indicated that the YANA-C, a former version of the HELENA-DIAT, showed good agreement with an interviewer-administered YANA-C interview. The HELENA-DIAT tool has been indicated as a good method to collect detailed dietary information from adolescents and was received well by the study participants [22]. Furthermore, a repeated 24-h recall was selected as the most suitable method to get population means and distributions by the European Consumption Survey Method (EFCOSUM) project [23]. To calculate energy and nutrient intake, data of the HELENA-DIAT was linked to the German Food Code and Nutrient Data Base (BLS (Bundeslebensmittelschlüssel), version II.3.1, 2005).

The usual dietary intake of nutrients and foods, also including episodically consumed foods, was estimated by the Multiple Source Method (MSM) https://nugo.dife.de/msm/[24]. The MSM calculates dietary intake for individuals first and then constructs the population distribution based on the individual data. With this method dietary data was corrected for between and within person variability. After the MSM was applied, dietary data were analyzed for average energy intake in kilocalories (kcal) and energy percentages (En%) from carbohydrates (CH), saccharides (monosaccharides & disaccharides), polysaccharides, proteins, fat, simple unsaturated fatty acids (SUFA), multiple unsaturated fatty acids (MUFA) and saturated fatty acids (SFA). Dietary cholesterol, dietary fiber, water, vitamin C, calcium and iron were also examined after they were adjusted for energy intake, using the nutrient density method (unit per 1000 kcal) [25]. No cases were excluded because of extreme over or underreported energy intake. In addition, daily servings of several food groups (g) were analyzed (see Table 1).

**Table 1:** Overview of foods included in each food group, according to the HELENA-DIAT (24 h recall)

Food group	Foods included
Water	Water Coffee & Tea

Bread and cereals (BrCe)	Bread & rolls Cereals

Grains and potatoes (GrPo)	Startch roots, potatoes Flour Pasta, Rice & other cereals

Fruit	Fruits

Vegetables	Vegetables (excluding potatoes) Soups & bouillons

Milk and milk products (Milk)	White milk with buttermilk Yoghurt and fromage blanc Milk, yoghurt & soya beverages Other milk products Desserts and puddings milk/soya based

Cheese	Cheese

Meat, fish and meat products (MeFiVe)	Meat & Fish Pulses Meat substitutions and vegetarian products Eggs

Spread and preparation fat (Spread)	Vegetable oils Margarine and lipids of mixed origin

Remaining group of fast food and snacks (RestFF)	Chocolate & confectionary Other sugar products Butter & animal fat Sauces Creams Cake, pie, biscuits Savoury snacks Sugar, honey, jam and syrup

Remaining group of beverages (RestB)	Fruit and vegetable juices Carbonated/soft/isotonic drinks including non alcoholic wine and beer Beer Wine and cider Other alcoholic beverages

#### International Physical Activity Questionnaire for Adolescents (IPAQ-A)

To assess PA of the last 7 days, an adolescent-adapted version of the International Physical Activity Questionnaire (IPAQ) was used, namely the IPAQ-A. The IPAQ is a self-administered questionnaire and originally developed for adults between 15-69 years, assessing the different domains of PA (work, transport, house and garden and leisure time). The questionnaire has been validated as a reliable tool to assess PA in an adult population [26]. To adapt the questionnaire to the HELENA population, questions about PA at work were replaced by questions about PA at school. Furthermore, the item relating domestic and gardening PA was reduced to one question. Also, the order of PA intensities was changed, to avoid over reporting [27]. The time spent at walking was asked before the time spent at vigorous and moderate PA intensity (versus vigorous, moderate and walking in the original IPAQ). As part of a pilot study, a concurrent validation study on this instrument (IPAQ-A), found significant, modest correlations (±0.20) between PA reported in the questionnaire and PA measured with accelerometers [28]. Also a higher correlation in the older adolescents in comparison with the younger ones was revealed [28]. For the IPAQ-A, total minutes per week (min/week) were computed for MVPA, walking not included, based on the guidelines for data processing and analyses of the IPAQ http://www.ipaq.ki.se/ipaq.htm. Furthermore, MVPA scores were cleaned and truncated at reasonable and realistic levels based on previous research [29]. PA scores were truncated in the different domains (school: max 1800 min/week or about 4 hr/day; home: max 1680 min/week or 4 hr/day; transport: max 1290 min/week or 3 hr/day; leisure time: max 1680 min/week or 4 hr/day; total PA: max 2540 min/week or about 6 hr/day) as well as in the different intensity levels (max 1260 min/week or 3 hr/day for moderate and vigorous PA). The PA levels were then categorized according to the tertiles in this study population.

#### Anthropometric measurements

Weight and height of the adolescents were measured by trained researchers in a standardized way [30]. Weight was recorded in underwear and without shoes to the nearest 0.1 kg, using an electronic scale (Type SECA 861). Height was measured barefoot in the Frankfort horizontal plane to the nearest 0.1 cm, using a telescopic height measuring instrument (Type SECA 225). Body Mass Index (BMI) of the adolescents was calculated as body weight in kg divided by the square of height in meters. The corresponding BMI z-scores, relative to the British 1990 Growth Chart References, were determined in order to obtain comparable values across both sexes and all ages [31]. The BMI z-score is the number of standard deviation units that a person's BMI deviates from a mean or reference value.

### Participants

In total, 3528 adolescents were recruited within the HELENA study. Of those, 2511 completed at least 75% of the IPAQ-A and filled in the HELENA-DIAT for at least two days. Crete could not be include in the 24-h dietary recall analyses since only a minority of the study population completed two 24-h recall days. Hungary could also not be included since no nutrient information was available. Therefore, only 8 study centers could be included for the 24-h dietary recall analyses (Stockholm, Dortmund, Ghent, Lille, Athens, Rome and Zaragoza) and finally, 2176 cases (46.2% male) remained eligible for further analyses. The excluded group was equally distributed for sex (50.1% male). A higher percentage of adolescents with high educated parents was seen in the included compared to the excluded sample (72.2% and 65.6% respectively). No differences were found between the included and excluded group for age, 14.7 years (SD 1.24) and 14.7 years (SD 1.20) respectively, and BMI, 21.1 (SD 3.65) and 21.7 (SD 3.84) respectively.

### Data analyses

The Statistical Package for the Social Sciences for Windows version 15 (SPSS Inc., Chicago, IL, USA) was used for data management and analyses. Descriptive statistics, including means, standard deviations (SD) or standard errors (SE) were calculated for each variable.

To compare the energy and nutrient intake and the food consumption between groups of adolescents with different PA levels (1^st ^to 3^rd ^tertile), analyses of covariance (ANCOVA) was used. The analyses were stratified for sex and controlled for age, BMI z-score, parental education which is defined by the highest level of education of the mother or father (low: lower education and lower secondary; high: higher secondary and higher education/university degree), study centre region (North: Stockholm; Central: Dortmund, Ghent, Lille; South: Athens, Rome, Zaragoza) and tanner stage (pubertal stage). To balance between type I and type II errors, an alpha level of p < 0.05 was used to decide upon statistical significance. Furthermore, a Bonferroni post-hoc test was conducted to make pair wise comparisons between the different PA tertiles.

## Results

Descriptive information (parental education level, tanner stage, region, age, BMI and time spent in MVPA) of the study sample can be found in Table 2. Age and total minutes spent in MVPA per week seemed to differ between the PA levels. In both sexes, those spending least time in MVPA were significantly older than those spending more time in MVPA. No differences could be found for BMI among the three PA levels.

**Table 2:** Descriptive statistics (parental education, tanner stage, region, age, BMI and time spent in MVPA) of the study sample.

	Physical activity level male			Physical activity level female		
	
	Tertile 1	Tertile 2	Tertile 3	Tertile 1	Tertile 2	Tertile 3
**Parental education level; N (%)**						
- Low	83 (25.0)	74 (21.8)	85 (25.4)	99 (25.6)	83 (21.3)	92 (23.3)
- High	235 (70.8)	251 (74.0)	239 (71.6)	273 (70.7)	292 (74.9)	287 (72.7)

**Tanner stage; N (%)**						
- Stage 1	2 (0.6)	1 (0.3)	6 (1.8)	0 (0.0)	0 (0.0)	0 (0.0)
- Stage 2	30 (9.0)	29 (8.6)	36 (10.8)	7 (1.8)	21 (5.4)	25 (6.3)
- Stage 3	58 (17.5)	80 (23.6)	100 (29.9)	71 (18.4)	80 (20.5)	127 (32.2)
- Stage 4	148 (44.6)	124 (28.3)	127 (38.0)	153 (39.6)	167 (42.8)	178 (45.1)
- Stage 5	79 (23.8)	96 (28.3)	56 (16.8)	133 (34.5)	112 (28.7)	58 (14.7)

**Region; N (%)**						
- North	34 (10.2)	25 (7.4)	42 (12.6)	46 (11.9)	63 (16.2)	56 (14.2)
- Centre	132 (39.8)	163 (48.1)	147 (44.0)	132 (34.2)	155 (39.7)	150 (38.0)

- South	130 (39.2)	112 (33.0)	76 (22.8)	179 (46.4)	128 (32.8)	96 (24.3)
**Age (yrs); mean (SD)**	15.0 (1.26)^a,b^	14.7 (1.26)	14.5 (1.21)	15.1 (1.19)^a,b^	14.7 (1.22)^c^	14.4 (1.16)
**BMI (kg/m^2^); mean (SD)**	21.6 (4.10)	21.1 (3.75)	21.1 (3.72)	21.4 (3.67)	21.1 (3.40)	21.0 (3.28)
**MVPA (min/week); mean (SD)**	224 (134.2)^a,b^	673 (146.9)^c^	1533 (429.8)	159 (90.1)^a,b^	487 (116.4)^c^	1248 (396.3)

MVPA: moderate to vigorous physical activity

^a ^Significant difference between 1^st ^and 2^nd ^tertile (Bonferroni post-hoc test)

^b ^Significant difference between 1^st ^and 3^rd ^tertile (Bonferroni post-hoc test)

^c ^Significant difference between 2^nd ^and 3^rd ^tertile (Bonferroni post-hoc test)

Table 3 and 4 show the results of the ANCOVA, stratified for sex, for self-reported PA levels and daily intake of energy and nutrients and food groups respectively. Table 3 shows that the mean daily energy intake did not differ significantly between the three PA levels in both sex groups. For the intake of the nutrients, differences could be found for the intake of saccharides, polysaccharides, protein, water and vitamin C between the PA levels in the male group. The least active males (1^st ^tertile) consumed less vitamin C and water compared to the most active ones (3^rd ^tertile). For water also a higher intake was seen in the most active males (3^rd ^tertile) compared to those of tertile 2. The intake of protein only differed between adolescents of tertile 2 and 3, with those of tertile 3 showing the highest intake. The most active males (3^rd ^tertile) also showed a lower intake of saccharides and a higher intake of polysaccharides compared to the least active ones (1^st ^tertile). For the polysaccharides, a difference was also seen between the males of tertile 1 and 2. In the female group, only a difference could be found for the intake of polysaccharides. Females with the highest physical activity level (3^rd ^tertile) consumed more polysaccharides compared to their least active peers (1^st ^tertile).

**Table 3:** Results of analyses of covariance of physical activity levels and daily intake of energy and nutrients^1,2^

		Physical activity level males					Physical activity level females				
							
		Tertile 1	Tertile 2	Tertile 3			Tertile 1	Tertile 2	Tertile 3		
		
		Mean (SE)	Mean (SE)	Mean (SE)	F	p	Mean (SE)	Mean (SE)	Mean (SE)	F	p
	Energy (kcal)	2469 (80.3)	2424 (82.5)	2469 (79.7)	0.27	0.765	1858 (42.1)	1873 (40.1)	1886 (41.8)	0.16	0.853

	CH (%En)	48.5 (0.69)	48.8 (0.70)	48.5 (0.68)	0.14	0.866	48.8 (0.50)	49.2 (0.48)	49.2 (0.50)	0.36	0.699
	- Saccharides	25.8 (0.59)^b^	24.6 (0.60)	24.3 (0.58)	4.50	0.014	25.1 (0.42)	25.0 (0.40)	24.3 (0.42)	1.65	0.194
	- Polysaccharides	21.7 (0.64)^a,b^	23.4 (0.66)	23.6 (0.63)	6.74	0.001	23.5 (0.48)^b^	24.1 (0.45)	25.0 (0.47)	3.53	0.030
	Protein (%En)	16.9 (0.31)	16.7 (0.32)^c^	17.5 (0.31)	4.69	0.009	16.4 (0.23)	16.4 (0.21)	16.4 (0.22)	0.01	0.999
	Fat (%En)	33.1 (0.60)	33.3 (0.61)	32.4 (0.59)	1.39	0.249	34.1 (0.43)	33.7 (0.41)	33.5 (0.43)	0.64	0.525
**Nutrients**	- SUFA	12.5 (0.25)	12.4 (0.26)	12.2 (0.25)	0.75	0.474	12.8 (0.19)	12.5 (0.18)	12.3 (0.19)	2.43	0.089
	- MUFA	4.5 (0.13)	4.6 (0.13)	4.6 (0.13)	0.53	0.589	5.0 (0.10)	4.9 (0.10)	4.9 (0.10)	0.20	0.816
	- SFA	13.6 (0.29)	13.7 (0.30)	13.2 (0.29)	2.23	0.109	13.9 (0.21)	13.8 (0.20)	13.9 (0.21)	0.17	0.844
	Cholesterol (g/1000 kcal)	157.5 (4.62)	153.5 (4.75)	160.9 (4.59)	1.56	0.211	158.0 (3.48)	159.1 (3.31)	163.9 (3.45)	0.19	0.306
	Dietary fiber	7.9 (0.20)	7.9 (0.21)	8.0 (0.20)	0.01	0.988	9.0 (0.17)	9.1 (0.16)	9.1 (0.17)	0.03	0.969
	(g/1000 kcal)	867 (27.9)^b^	878 (28.6)^c^	960 (27.7)	7.57	0.001	953 (24.6)	1003 (23.4)	1014 (24.4)	2.57	0.077
	Water (g/1000 kcal)	38.7 (2.45)^b^	42.3 (2.52)	46.7 (2.44)	6.00	0.003	49.2 (2.09)	51.0 (1.99)	50.6 (2.08)	0.30	0.742
	Vit.C (mg/1000 kcal)	449.2 (12.8)	469.7 (13.2)	461.0 (12.7)	1.65	0.193	429.8 (9.54)	444.6 (9.07)	440.5 (9.47)	1.02	0.363
	Calcium (mg/1000 kcal)	5.7 (0.12)	5.6 (0.12)	5.7 (0.12)	0.45	0.636	5.8 (0.10)	5.7 (0.10)	5.9 (0.10)	0.98	0.377
	Iron (mg/1000 kcal)										

^1 ^data are stratified for sex (Males N = 1005; Females N = 1171)

^2 ^analyses are controlled for age, BMI z-score, parental education level, study centre region and Tanner stage

CH = carbohydrates

SUFA = Simple unsaturated fatty acids

MUFA = Multiple unsaturated fatty acids

SFA = Saturated fatty acids

^a ^Significant difference between 1^st ^and 2^nd ^tertile

^b ^Significant difference between 1^st ^and 3^rd ^tertile

^c ^Significant difference between 2^nd ^and 3^rd ^tertile

**Table 4:** Results of analyses of covariance of physical activity levels and daily intake of foods^1,2^

	Physical activity level males					Physical activity level females				
						
	Tertile 1	Tertile 2	Tertile 3			Tertile 1	Tertile 2	Tertile 3		
	
	Mean (SE)	Mean (SE)	Mean (SE)	F	p	Mean (SE)	Mean (SE)	Mean (SE)	F	p
Water (g)	680 (50.6)	606 (52.0)	687 (50.6)	2.01	0.134	708 (34.5)	736 (32.8)	740 (34.2)	0.38	0.687
BrCe (g)	129.8 (6.07)	121.9 (6.23)	121.4 (6.02)	1.45	0.236	101.9 (3.69)^b^	97.4 (3.51)	91.2 (3.66)	3.09	0.046
GrPo (g)	216.4 (7.94)^a^	199.3 (8.15)	204.1 (7.88)	3.12	0.045	174.2 (4.81)	164.3 (4.57)	161.5 (4.77)	2.90	0.055
Fruit (g)	101.3 (10.00)^b^	112.0 (10.27)^c^	134.6 (9.92)	6.59	0.001	111.1 (6.39)	127.8 (6.08)	126.1 (6.34)	3.20	0.041
Vegetables (g)	131.5 (8.61)	148.5 (8.85)	147.8 (8.55)	3.02	0.049	122.0 (5.66)	124.4 (5.38)	123.3 (5.62)	0.08	0.928
Milk (g)	353.4 (21.79)	397.7 (22.39)	396.9 (21.64)	3.28	0.038	221.9 (12.11)^a,b^	257.8 (11.51)	273.6 (12.01)	7.08	0.001
Cheese (g)	34.0 (2.34)^a,b^	28.4 (2.41)	26.2 (2.33)	6.90	0.001	29.1 (1.49)^b^	26.2 (1.41)	24.6 (1.48)	3.38	0.035
MeFiVe (g)	213.8 (8.85)^b^	209.9 (9.09)^c^	230.1 (8.79)	3.44	0.032	174.3 (5.12)	178.6 (4.86)	175.8 (5.08)	0.28	0.747
Spread (g)	15.2 (1.35)	14.6 (1.39)	13.1 (1.34)	1.38	0.252	11.1 (0.66)^a,b^	8.3 (0.63)	7.8 (0.66)	10.73	<0.001
RestFF (g)	139.5 (6.76)	136.5 (6.94)	134.1 (6.71)	0.36	0.700	152.9 (4.29)	127.5 (4.08)	131.7 (4.25)	2.38	0.093
RestB (g)	515.8 (34.43)	510.0 (35.36)	556.9 (34.18)	1.23	0.293	345.6 (17.41)	3367.6 (16.56)	341.9 (17.28)	0.09	0.917

^1 ^data are stratified for sex (Males N = 1005; Females N = 1171)

^2 ^analyses are controlled for age, BMI z-score, parental education level, study centre region and Tanner stage

BrCe = Bread and cereals

GrPo = Grain products and potatoes

MeFiVe = Meat, fish, eggs, meat substitutes and vegetarian products

RestFF = Fast Food from Remaining group

RestB = Beverages from Remaining group

^a ^Significant difference between 1^st ^and 2^nd ^tertile

^b ^Significant difference between 1^st ^and 3^rd ^tertile

^c ^Significant difference between 2^nd ^and 3^rd ^tertile

When looking at the food groups (Table 4), the consumption of fruit, milk products and cheese seemed to differ significantly between the PA levels in both sexes. For fruit and cheese, the difference was greater in the male group. The Bonferroni post-hoc test showed a significant higher intake of fruit by the most active males (3^rd ^tertile) compared to the least active (1^st ^tertile) and those from tertile 2. The same trend could be observed in females, but the post hoc tests were not significant. The least active males (1^st ^tertile) also reported to eat more cheese compared to those from tertile 2 and 3, whereas only a difference between tertile 1 and 3 could be found in the female group. The difference in milk consumption was greater in the female group. The Bonferroni post-hoc test showed a significant lower intake of milk products by the least active females (1^st ^tertile) compared to the most active (3^rd ^tertile) and those from tertile 2. For the males, the post hoc tests were not significant. A difference in the consumption of grain products and potatoes, vegetables and meat, fish, eggs, meat substitutes and vegetarian products was also reported in the male group. The least active males (1^st ^tertile) consumed more grain and potatoes than those from tertile 2, while the the males spending most time in MVPA (3^rd ^tertile) reported to eat more meat, fish, eggs, meat substitutes and vegetarian products compared to the those from tertile 1 and 2. The females spending less time in MVPA (1^st ^tertile), reported to consume most spreads compared to females in the other two PA levels (2^nd ^and 3^rd ^tertile). The Bonferroni post-hoc test also revealed that females in the highest PA level (3^rd ^tertile) reported to consume less bread and cereal products than females in the lowest PA level (1^st ^tertile).

In both the male and female adolescents, no differences in the consumption of products from the remaining food groups could be found between the levels of PA.

## Discussion

In both, male and female adolescents, the energy intake did not differ significantly between the three PA levels. Cavadini et al. [32] found, in a small subsample of 11-15 year old adolescents, that the energy intake was higher among those who participated more frequently in sport activities, but this trend also seemed statistically non-significant. Croll et al. [33] revealed a higher mean daily energy intake among sport-involved males compared to non-sport-involved males, whereas no difference could be found in the female group. In a study with African-American girls, the total caloric intake was also not significantly associated with PA [34]. Not finding any univocal results between those studies and the present one, could be explained by the fact that energy expenditure from physical activity is only a part of the total energy expenditure. It is noteworthy that longitudinal studies are necessary to examine the energy balance among adolescents in more depth. In addition, sedentary lifestyles have recently been linked to a higher food intake and should therefore be kept in mind when estimating the energy requirements. As sedentarism cannot be seen as the opposite of physical activity, it is possible that a highly physically active person, also shows more sedentary behaviors by means of compensation [35]. Furthermore, the physical activity induced energy expenditure is a variable factor in the energy balance equation. The level of physical activity can vary from day to day within a person. Adaptations in the level of physical activity does not always result in immediate changes in energy intake, in other words energy intake and energy expenditure do not balance on a daily basis [36].

Concerning the food intake, both in the male and female group, significant differences were found for the consumption of fruit, milk products and cheese. A higher consumption of fruit and milk products was reported by the most active adolescents compared to the least active ones. On the other hand, adolescents reporting to spent more time in MVPA, consumed less cheese compared to those spending less time in MVPA. The higher fruit and milk consumption within the more active adolescents is in line with previous studies [16,32], which observed positive associations between the consumption of fruit and dairy products and higher physical activity levels in both boys and girls.

In the female group, a higher consumption of bread and cereal products was found for those spending less time in MVPA compared to their more active peers. For males, the intake of those products was similar between the different PA levels. In a study by Cavadini et al. [32], no difference in bread consumption between athletic and non-athletic adolescents was shown. In addition they observed higher cereal consumption in the more active group, especially among girls, which is in discrepancy with our findings. In the present study, bread and cereals were seen as one food group, whereas Cavadini et al. [32] examined the intake of bread and cereals separately and this could be an explanation for the inconsistent results between both studies.

Furthermore, the consumption of products from the remaining food groups did not differ between the levels of PA. Previous research also revealed no relationship between the time spent in MVPA and the frequency of snacking and intake of sugared beverages [16,32,34]. The above mentioned findings concerning the food consumption, indicate that spending more time in physical activities does not exclusively result in healthier eating habits. While some studies suggest that active people are inclined to eat healthier diets [18], there are also a lot of discrepancies found in the literature examining the correlations between multiple health behaviors [12].

Next to the differences found in the intake of the above mentioned food groups between the PA levels, differences in some nutrient intakes could be found. The more active male adolescents showed a higher intake of polysaccharides, protein, water and vitamin C. On the other hand, a lower intake of saccharides was observed in those adolescents. The higher water and vitamin C intake could be explained by the higher consumption of fruit and vegetables, which are known to be rich of those nutrients. The higher consumption of meat, fish, eggs, meat substitutes and vegetarian products can be the reason for the higher protein intake observed in the more active males. The differences found for the saccharides and polysaccharides is difficult to explain. Fruit is a source of saccharides, whereas vegetables and grains and potatoes provide more polysaccharides. This is in contrast with the differences found between those nutrients. A possible explanation for not finding parallel differences in nutrient intake with differences in food group intake, could be that the consumption of different foods, compensate the nutrient intake. A higher consumption of one food product, but a lower intake of another, could stabilize the nutrient intake, especially on population level. Also, a lack of meaningful difference in the amount of foods consumed could be an explanation for not finding more significant differences in the intake of nutrients among the physical activity levels. For example, the range of difference in gram for vegetables for males among the 3 PA levels was only 17 gram and the difference for spreads in the female group was only 3.3 gram. More detailed research is necessary to examine the relation between the nutrient and food intake. On the other hand, not finding more significant differences in nutrient intake between the more active and less active adolescents, is again an indication for the fact that physical activity is not automatically correlated with healthier diets.

When interpreting the results of the present study we have to keep in mind some limitations. The instruments to assess dietary intake and PA were self-reporting questionnaires and those have their well known disadvantages. The limitations of those instruments could cause inaccuracy in the time spent in PA and the nutrient and food intake assessed. In fact, misreporting is a common problem in assessing dietary habits and PA in adolescents [37,38]. Evidence for under-reporting of food intake with self-reported dietary assessments has been revealed previously, especially in girls [39]. It also appears that the food intake data of most adolescents are particularly prone to reporting error, also at the group level [40]. Furthermore 2 days of 24 h recall are scientifically not ideal, although more days were practically not possible. Therefore the dietary intake data has been corrected for within and between person variability according to the MSM method [24], so the reporting error could be partly counteracted. Furthermore, it is most likely that the reported minutes of MVPA are not equal to the total energy expenditure, which also has to be linked to other factors like BMR (basal metabolic rate), growth, etc. On the other hand, both questionnaires have been tested and validated before use in the HELENA study [21,22,28]. It should also be mentioned that the study population is an urban population and that extrapolation to a general European population should be treated cautiously. Strengths of the present study are the large sample size and diverse geographical origin in Europe. Also, the highly standardized procedures used within the HELENA study are an important strength. In addition, this study is one of the first in examining the association between dietary intake, on both the nutrient and food group level, and PA levels among European adolescents.

## Conclusion

From the present results it can be concluded that dietary habits diverge between adolescents with different self-reported PA levels. For some food groups a significant difference in intake could be found between the more active and less active adolescents. Those differences found, were also reflected in differences in some nutrient intakes. Furthermore, adolescents within each PA level, reported an equal consumption of foods from the remaining food group. Therefore, the present findings are an indication that the more physically active adolescents are not always inclined to eat healthier diets than their less active peers. Because food habits and PA, have both been associated with several chronic diseases [1,2], both behaviors should be taken into account when examining health and development of those diseases. Optimizing the diet quality in both the inactive and active adolescents and increasing the time spending in MVPA in those with low PA levels still remain necessary in interventions.

## Competing interests

The authors declare that they have no competing interests.

## Authors' contributions

CO participated in the design of the study, data collection, analysis, interpretation of the results and drafted the manuscript. IH was involved in manuscript drafting and coordinated the statistical analysis. IH, IDB, SDH and MH contributed to the results interpretation, and editing of the manuscript. LAM coordinated the total HELENA study on international level. LAM, FG, AK, MS and KW were involved in the design of the HELENA study and locally coordinated the project. CO, LB, MMC and CLD performed the data collection locally. All authors participated in the writing of the paper and provided comments on the drafts and approved the final version.

## References

[B1] Warburton DER Nicol CW Bredin SSD Health benefits of physical activity: the evidence Can Med Assoc J 2006 174 801 809 10.1503/cmaj.051351 PMC1402378 16534088

[B2] World Health Organization Diet, Nutrition and the Prevention of Chronic Diseases. WHO/FAO Expert Consultation.: 916 2002 Geneva: WHO

[B3] Maffeis C Aetiology of overweight and obesity in children and adolescents Eur J Pediatr 2000 159 S35 S44 10.1007/PL00014361 11011954

[B4] Singh AS Mulder C Twisk JWR Van Mechelen W Chinapaw MJM Tracking of childhood overweight into adulthood: a systematic review of the literature Obes Rev 2008 9 474 488 10.1111/j.1467-789X.2008.00475.x 18331423

[B5] Lake AA Mathers JC Rugg-Gunn AJ Adamson AJ Longitudinal change in food habits between adolescence (11-12 years) and adulthood (32-33 years): the ASH30 study J Public Health-UK 2006 28 10 16 10.1093/pubmed/fdi082 16473923

[B6] Telama R Tracking of Physical Activity from Childhood to Adulthood: A Review Obesity Facts 2009 2 187 195 10.1159/000222244 20054224 PMC6516203

[B7] Currie C Gabhainn SN Godeau E Roberts C Smith R Currie D Picket W Richter M Morgan A Barnekow V Inequalties in young people's health: HBSC internaional report from 2005/2006 survey: Health Policy for Children and Adolescents 2008 5 World Health Organization Regional Office for Europe 208

[B8] Biddle SJH Gorely T Stensel DJ Health-enhancing physical activity and sedentary behaviour in children and adolescents J Sports Sci 2004 22 679 701 10.1080/02640410410001712412 15370482

[B9] Riddoch CJ Andersen LB Wedderkopp N Harro M Klasson-Heggebo L Sardinha LB Cooper AR Ekelund U Physical activity levels and patterns of 9-and 15-yr-old European children Med Sci Sports Exerc 2004 36 86 92 10.1249/01.MSS.0000106174.43932.92 14707773

[B10] Currie C Roberts C Morgan A Smith R Settertobulte W Samdaland O Rasmussen VB Health Behaviour in School-aged Children (HBSC) study: international report from the 2001/2002 survey: Health Policy for Children and Adolescents 2004 4 World Health Organization Regional Office for Europe 237

[B11] Pronk NP Anderson LH Crain AL Martinson BC O'Connor PJ Sherwood NE Whitebird RR Meeting recommendations for multiple healthy lifestyle factors-Prevalence, clustering, and predictors among adolescent, adult, and senior health plan members Am J Prev Med 2004 27 25 33 10.1016/j.amepre.2004.04.022 15275671

[B12] Kremers SPJ De Bruijn GJ Schaalma H Brug J Clustering of energy balance-related behaviours and their intrapersonal determinants Psychol Health 2004 19 595 606 10.1080/08870440412331279630

[B13] Pearson N Atkin AJ Biddle SJH Gorely T Edwardson C Patterns of adolescent physical activity and dietary behaviours Int J Behav Nutr Phys Act 2009 6:45 10.1186/1479-5868-6-45 19624822 PMC2718856

[B14] Driskell MM Dyment S Mauriello L Castle P Shennan K Relationships among multiple behaviors for childhood and adolescent obesity prevention Prev Med 2008 46 209 215 10.1016/j.ypmed.2007.07.028 17714771

[B15] Department of Health Choosing Health: Making healthy choices easier 2004

[B16] Kelishadi R Ardalan G Gheiratmand R Gouya MM Razaghi EM Delavari A Majdzadeh R Heshmat R Motaghian M Bareketi H Mahmoud-Arabi MS Riazi MM Association of physical activity and dietary behaviours in relation to the body mass index in a national sample of Iranian children and adolescents: CASPIAN Study B World Health Organ 2007 85 19 26 10.2471/BLT.06.030783 PMC2636217 17242754

[B17] Coulson NS Eiser C Eiser JR Diet, smoking and exercise: Interrelationships between adolescent health behaviours Child Care Hlth Dev 1997 23 207 216 10.1111/j.1365-2214.1997.tb00964.x 9158910

[B18] Elder SJ Roberts SB The effects of exercise on food intake and body fatness: A summary of published studies Nutr Rev 2007 65 1 19 10.1111/j.1753-4887.2007.tb00263.x 17310855

[B19] Moreno LA Gonzalez-Gross M Kersting M Molnar D De Henauw S Beghin L Sjostrom M Hagstromer M Manios Y Gilbert CC Ortega FB Dallongeville J Arcella D Warnberg J Hallberg M Fredriksson H Maes L Widhalm K Kafatos AG Marcos A Assessing, understanding and modifying nutritional status, eating habits and physical activity in European adolescents: The HELENA (Healthy Lifestyle in Europe by Nutrition in Adolescence) Study Public Health Nutr 2008 11 288 299 10.1017/S1368980007000535 17617932

[B20] Beghin L Castera M Manios Y Gilbert CC Kersting M De Henauw S Kafatos A Gottrand F Molnar D Sjostrom M Leclercq C Widhalm K Mesana MI Moreno LA Libersa C Quality assurance of ethical issues and regulatory aspects relating to good clinical practices in the HELENA Cross-Sectional Study Int J Obes 2008 32 S12 S18 10.1038/ijo.2008.179 19011647

[B21] Vereecken CA Covents M Sichert-Hellert W Alvira JMF Le Donne C De Henauw S De Vriendt T Phillipp MK Beghin L Manios Y Hallstrom L Poortvliet E Matthys C Plada M Nagy E Moreno LA Development and evaluation of a self-administered computerized 24-h dietary recall method for adolescents in Europe Int J Obes 2008 32 S26 S34 10.1038/ijo.2008.180 19011650

[B22] Vereecken CA Covents M Matthys C Maes L Young adolescents' nutrition assessment on computer (YANA-C) Eur J Clin Nutr 2005 59 658 667 10.1038/sj.ejcn.1602124 15741983

[B23] Brussaard JH Lowik MRH Steingrimsdottir L Moller A Kearney J De Henauw S Becker W A European food consumption survey method-conclusions and recommendations Eur J Clin Nutr 2002 56 S89 S94 10.1038/sj.ejcn.1601432 12082521

[B24] Haubrock J Harttig U Souverein O Boeing H An improved statistical tool for estimating usual intake distributions: the Multiple Source Method (MSM) Archives of public Health 2010 68 14 15

[B25] Willet WC Nutritional Epidemiology 1998 2 New York; Oxford University Press

[B26] Craig CL Marshall AL Sjostrom M Bauman AE Booth ML Ainsworth BE Pratt M Ekelund U Yngve A Sallis JF Oja P International physical activity questionnaire: 12-country reliability and validity Med Sci Sports Exerc 2003 35 1381 1395 10.1249/01.MSS.0000078924.61453.FB 12900694

[B27] Barnett J Nigg CR De Bourdeaudhuij I Maglione C Maddock J The effect of item order on physical activity estimates using the IPAQ Californian J Health Promot 2007 5 23 29

[B28] Hagstromer M Bergman P De Bourdeaudhuij I Ortega FB Ruiz JR Manios Y Rey-Lopez JP Phillipp K von Berlepsch J Sjostrom M Concurrent validity of a modified version of the International Physical Activity Questionnaire (IPAQ-A) in European adolescents: The HELENA Study Int J Obes 2008 32 S42 S48 10.1038/ijo.2008.182 19011653

[B29] Haerens L Deforche B Maes L Cardon G De Bourdeaudhuij I Physical activity and endurance in normal weight versus overweight boys and girls J Sports Med Phys Fitness 2007 47 344 350 17641603

[B30] Nagy E Vicente-Rodriguez G Manios Y Beghin L Iliescu C Censi L Dietrich S Ortega FB De Vriendt T Plada M Moreno LA Molnar D Harmonization process and reliability assessment of anthropometric measurements in a multicenter study in adolescents Int J Obes 2008 32 S58 S65 10.1038/ijo.2008.184 19011655

[B31] Cole TJ Freeman JV Preece MA Body-Mass Index Reference Curves for the Uk, 1990 Arch Dis Child 1995 73 25 29 10.1136/adc.73.1.25 7639544 PMC1511150

[B32] Cavadini C Decarli B Grin J Narring F Michaud PA Food habits and sport activity during adolescence: differences between athletic and non-athletic teenagers in Switzerland Eur J Clin Nutr 2000 54 S16 S20 10805033 10.1038/sj.ejcn.1600979

[B33] Croll JK Neumark-Sztainer D Story M Wall M Perry C Harnack L Adolescents involved in weight-related and power team sports have better eating patterns and nutrient intakes than non-sport-involved adolescents J Am Diet Assoc 2006 106 709 717 10.1016/j.jada.2006.02.010 16647329

[B34] Jago R Baranowski T Yoo S Cullen KW Zakeri I Watson K Himes JH Pratt C Sun WJ Pruitt LA Matheson DM Relationship between physical activity and diet among African-American girls Obesity Research 2004 12 55S 63S 10.1038/oby.2004.269 15489468

[B35] Jackson DM Djafarian K Stewart J Speakman JR Increased television viewing is associated with elevated body fatness but not with lower total energy expenditure in children Am J Clin Nutr 2009 89 1031 1036 10.3945/ajcn.2008.26746 19244374

[B36] Westerterp KR Physical activity, food intake, and body weight regulation: insights from doubly labeled water studies Nutrition Reviews 2010 68 148 154 10.1111/j.1753-4887.2010.00270.x 20384845

[B37] Armstrong N Welsman JR The physical activity patterns of European youth with reference to methods of assessment Sports Med 2006 36 1067 1086 10.2165/00007256-200636120-00005 17123328

[B38] Moreno LA Kersting M De Henauw S Gonzalez-Gross M Sichert-Hellert W Matthys C Mesana MI Ross N How to measure dietary intake and food habits in adolescence: the European perspective Int J Obes 2005 29 S66 S77 10.1038/sj.ijo.0803063 16385756

[B39] Dodd CJ Energy regulation in young people J Sport Sci Med 2007 6 327 336 PMC3787283 24149419

[B40] Livingstone MBE Robson PJ Wallace JMW Issues in dietary intake assessment of children and adolescents Brit J Nutr 2004 92 S213 S222 10.1079/BJN20041169 15522159

